# Utilizing the spirituality of funeral rituals for post-pandemic grief recovery

**DOI:** 10.3389/fpsyg.2022.1040482

**Published:** 2023-01-05

**Authors:** Carl B. Becker

**Affiliations:** Policy Science Unit, Kyoto University, Kyoto, Japan

**Keywords:** death, mortality, bereavement, social capital, meaning, mourning, religion, tradition

## Introduction

Well over 15 million people have died directly or indirectly from the pandemic, not only from COVID-19, but also from excess deaths—diseases and injuries receiving inadequate care due to COVID-19 restrictions and hospital overload (WHO News, 2022). The pandemic and its excess deaths have bereaved tens of millions, including probably 10 million children (Hillis et al., 2022) and 7 million caregivers, raising their risks of disease, trauma, and mental health issues (Bovero et al., 2022). These bereaved face higher risks of morbidity and mortality (Seiler et al., 2020); some 10–20% of all bereaved face psychological and physical complications of grief that require medical intervention in the years following their bereavement (Aoun et al., 2015; Jadhav and Weir, 2017). In countries with public health plans, this means that grief and bereavement are not only private psycho-spiritual problems, but also constitute a drain on public funds and medical resources.

Traditionally, funerals and rituals addressed and ameliorated some of that grief (see Hoy, 2013, 2020), but pandemic restrictions have hampered funeral gatherings as well. Research suggests that funerals and related rituals contribute substantially to the psycho-spiritual as well as physical health of the recently bereaved (Becker et al., 2021a,b) yet ironically, the psycho-spiritual value of funerals has not yet been widely studied nor applied to prevent or address psychiatric problems. This essay would propose that funerary rituals support the bereaved by facilitating (1) expression; (2) solace; (3) support; and (4) meaning-making, all central to the spiritual wellbeing of the bereaved.

## Enabling *expression* of thanks or farewell as well as grief to the spirit of the departed

Funerals provide a psycho-social space in which otherwise socially prohibited emotion can be expressed, whether by dramatic overt wailing as in Korea or Samoa, or by mourning suits, veils, and carefully-chosen phrases, as in the UK or Japan. In addition to granting recognition to the status and suffering of the bereaved, funerals also provide a chance to convey last words of gratitude, farewell, or *bon voyage*, which other factors may render impossible prior to the passing (Himonya, 1996, p. 30). When COVID-19 hospital restrictions prohibit families and relatives from visiting their dying loved ones, this chance to convene and honor the recently departed becomes unforgettably significant (Field and Filanosky, 2009; Mayland et al., 2021).

Mourners often *feel* the presence of the spirit of the deceased near their corpse, and begin the peaceful transition, not to forgetting but to reformulating their continuing bonds with the *spirit* of the deceased (Chan et al., 2005). While the idea of continuing bonds dates back to antiquity, it has been widely adopted and adapted throughout the scholarly world, leading scholars to observe that grief is not something to be overcome but something to live with and learn from (cf. Klass and Steffen, 2018). The psychological as well as physical space to express grief in socially acceptable ways, and to be allowed to feel and speak to the presence of the departed, whether at home altars, gravesites, or churches, may be salvific to otherwise distraught survivors.

## Finding spiritual *solace* through ritual, chanting, prayer, or meditation

When one's life-world crumbles into shambles at the loss of a breadwinner, parent, or beloved partner, then words, prayers, or songs known and treasured since childhood can provide a natural structure upon which to rebalance and rebuild, while taking on new and deeper meanings (Hoy, 2013, p. 11). In some cultures, familiar religious music or hymns enable funeral participants to feel and express solidarity when other language may not feel right (Adamson and Holloway, 2012a,b). While western religions may prioritize prayer to spiritual entities on behalf of the spirit of the deceased, some eastern traditions would advocate meditation to calm unsettled emotions and restore a more selfless equanimity; for many Japanese, simply listening to the chanting of Buddhist priests demonstrably lowers the stress hormones (and anguish!) of the bereaved (Taniyama et al., 2019). Such practices, particularly if familiar from childhood, can confer a solace deeper than mere words of condolence—yet another reason that children should be encouraged to attend funerals (cf. Schonfeld and Demaria, 2016; Søfting et al., 2016).

## Eliciting spiritual and social *support* from a community of friends and relations

While urban and rural gatherings show substantial variations (Zamfirache, 2021), funerals are often the last chance to re-connect with life-long but fading networks (cf. O'Rourke and Spitzberg, 2011). Not every friend or relative who is invited to a funeral will attend, nor will every participant readily devote themselves to helping the bereaved widow or family. But inviting only the immediate family virtually destroys any chance for broader social support. If people are only informed of a friend's or classmate's passing many months later, they receive not only the information of the decease, but also the sub-text that they were not considered close enough to invite. By contrast, if the funeral casts a broad net, informing and inviting all the relatives, friends, and classmates who might feel interested, then the self-selected significant ones will attend. Among them, those who feel closest or most concerned may subsequently offer to chat, dine, shop, travel, or do things together with the bereaved.

If even a few old friends or relations extend a hand to the bereaved after the funeral, their outreach is infinitely more natural and acceptable, not to mention cost-effective, than that of a social worker suddenly summoned to deal with the psychological or psychiatric troubles of another unknown elder. The social capital of friends and family convening at a funeral, who already know the character and spiritual proclivities of the bereaved, can provide emotional and spiritual as well as social support far beyond the provenance of tax-based social services or psychiatrists (Aoun et al., 2018). To be sure, pandemic containment measures may detrimentally restrict the number allowed to attend (Pellecchia et al., 2015), but funeral attendance and subsequent social support significantly protect against the continuing or complicated grief of family caregivers bereaved during pandemics (Bovero et al., 2022).

## Deriving healing and *meaning-making* through interaction with funeral directors

Death in the family frequently destroys their sense of meaning. Funeral directors' gentle engagement can begin the important process of reconstructing meaning (Neimeyer et al., 2006; Holloway et al., 2013). Typically, funeral directors are among the first professionals to encounter the bereaved shortly after they witness or learn of their loved one's death. In the first hours or days after bereavement, families waver helplessly between shock, disbelief, anger and sorrow. Sharing these most personal emotions can make the funeral director feel like an intimate friend or counselor. Funeral directors encounter unique “teachable moments” in which they can elicit the roles and meanings that the departed held for the family, helping them to commemorate that life and those values in perpetuity. Conversely, if the director's words or actions betray unconcern or mundane commercialism, any previously established intimacy can feel like crass betrayal.

If we take spirituality to mean not merely religiosity but also the expression of personal meaning and values, then the funeral director's involvement at this most sensitive time is a prime opportunity for spiritual healing. To be sure, not all funeral directors have adequate education in psychology or counseling, but the more successful ones develop interpersonal skills either through years of practice or supplementary coursework. Our research has encountered widowers who aver that the funeral director or staff saved them from suicide; conversely, dissatisfactory funerals correlate with continuing long-term grief and greater reliance on medicine and pharmaceuticals at public expense (Becker et al., 2022).

## Discussion

It is natural if not universal for survivors to feel the spirit of their loved ones for some time after their decease, and to conduct culturally appropriate rituals to commemorate the deceased (Metcalf and Huntington, 1991). To be sure, some cultures ritually preserve memories of their loved ones more formally and for longer periods than others. For example, it is common for Japanese to enshrine their ancestors in home altars, where they offer them fresh food daily, where monks visit monthly to chant ritual prayer services for the peace of their souls, and where family and friends may gather annually for two or three years after the death to remember the deceased (see Becker and Kashio, 2021). Throughout East Asia, families typically clean and decorate the graves of their ancestors at the vernal and autumnal equinoxes, and hold prayers or festivals for their ancestors at the solstices. Early research comparing Japanese widows' grief with those in Parkes' surveys suggested that Japanese daily rituals remembering the deceased (or their sensibility to the invisible presence of the deceased) made them more able to accept their losses—although not less depressed or sleepless (Yamamoto et al., 1969).

Ironically, modern Japanese have begun to forget the importance of funeral and memorial rituals. More recent research (Yamada and Suzuki, 2014) shows that social mobility, nuclear families, and secularization had begun to erode such rituals even before COVID-19. Today, the ongoing pandemic challenges not only virologists and epidemiologists but also those bereaved during pandemic-based prohibitions of large assemblies (see MacNeil et al., 2021). The combination of sudden unexpected death and loss of income due to economic downturn alone would predict more difficulty in accepting the death of a loved one. Yet COVID-19 also makes traditional funeral gatherings impossible. Is this merely a sentimental issue, or does the elimination of funeral gatherings affect the psychological and physical health of the bereaved?

Becker et al. (2021a,b) conducted Japan's first major nationwide survey to determine the impact of funeral and memorial practices on psychological grief, physical health, and medical costs. Their preliminary findings were that deeper grief after bereavement correlated with more physical problems and more medical dependency in Japan. High satisfaction with funerals loosely correlated with less expenditure of personal time and money for medical and psychological issues after bereavement, while funeral *dis*satisfaction correlated more strongly with heightened medical reliance—but they were unable to find statistically significant correlation between specific rituals or funeral practices and specific symptoms of grief (Becker et al., 2021b, 2022). In other words, funerals affect the bereaved not only emotionally and spiritually but also psycho-somatically.

While rituals surely take cultural forms, every culture uses rituals like funerals to accommodate the otherwise crushing grief at the loss of loved ones. The previous paragraphs highlight some effects of funerals and ongoing rituals in Japan, but the dimensions of funerals discussed here are by no means limited to East Asian contexts, and we should hope that similar research on their effects will be replicated in other cultures as well.

## Conclusion

As societies age, greater proportions of the population pass away naturally, yet their survivors are rarely psychologically or spiritually prepared for the loss of their beloved friends and elders. Recognizing the dangers of continuing grief, the DSM-5 and ICD-11 have included bereavement grief in their diagnostic categories. Shear's noteworthy programs (Shear et al., 2016) have proven clinically effective in addressing problematic grief. But the fact that many cultures continue to revere their dead for several years after their passing suggests that grief is not something to be “overcome” by clinical counseling or medication, but rather that it presents occasions for more humbly contemplating the transcendent spiritual aspects of our personalities or interpersonal interconnections.

Funerals alone cannot address every psycho-spiritual issue that death—especially sudden death—leaves in its wake. Yet funerals can initiate crucial emotional, cultural-religious, psycho-social, and meaning-restoring processes to restabilize the continuing lives of the bereaved. Along with the death of a loved one, the loved one's funeral can leave deep memories which impact the mental health of the bereaved over the following months and years. Since funeral directors and priests are among the first whom the bereaved encounter after their loss, their influence looms particularly strong. This article urges that professional psychologists and psychiatrists too should work more closely with traditional networks of family and cultural ritual for the spiritual wellbeing of our clients.

## Author contributions

The author confirms being the sole contributor of this work and has approved it for publication.
